# Money Matters: Time for Prevention and Early Intervention to Address Family Economic Circumstances

**DOI:** 10.1007/s10935-022-00717-9

**Published:** 2023-03-13

**Authors:** Nick Axford, Vashti Berry

**Affiliations:** 1 NIHR ARC South West Peninsula (PenARC), University of Plymouth, N10, ITTC Building, Plymouth Science Park, Plymouth, PL6 8BX UK; 2 NIHR ARC South West Peninsula (PenARC), University of Exeter, 2.05 South Cloisters, St. Luke’s Campus, Heavitree Road, Exeter, EX1 2LU UK

**Keywords:** Child, Early intervention, Financial, Hardship, Money, Poverty, Prevention, Youth

## Abstract

Child poverty is associated with poorer physical and mental health, negative educational outcomes and adverse long-term social and psychological consequences, all of which impact on service demand and expenditure. Until now, however, prevention and early intervention practice has tended to focus on enhancing inter-parental relationships and parenting skills (e.g., via relationship skills education, home visiting, parenting programs, family therapy) or child language, social-emotional and life skills (e.g., early childhood education, school-based programs, youth mentoring). Programs often target low-income neighborhoods or families but rarely address poverty *directly*. While there is substantial evidence for the effectiveness of such interventions in improving child outcomes, null results are not uncommon and even positive effects are often small, short-term, and difficult to replicate. One avenue to enhance intervention effectiveness is to improve families’ economic circumstances. There are several arguments for this refocusing. It is arguably unethical to focus on individual risk without acknowledging or seeking to address (where relevant) families’ social and economic contexts, while the stigma and material constraints associated with poverty can make it harder for families to engage with psychosocial support. There is also evidence that increasing household income improves child outcomes. Although national policies to alleviate poverty are important, it is increasingly recognized that practice-based initiatives have a role to play (e.g., income maximization, devolved budgets, money management support). However, knowledge about their implementation and effectiveness is relatively thin. For instance, there is some evidence that co-located welfare rights advice in healthcare settings can improve recipients’ financial circumstances and health, but it is mixed and of limited quality. Moreover, there is little rigorous research on whether and how such services affect mediators (parent-child interactions, parenting capacity) and/or child physical and psychosocial outcomes directly. We call for prevention and early intervention programs to attend more to families’ economic circumstances, and for experimental studies to test their implementation, reach and effectiveness.

## Introduction

The need to improve child outcomes is widely acknowledged, especially following the COVID-19 pandemic. School closures and social lockdowns were associated with adverse mental health symptoms (e.g., distress, anxiety) and health behaviors (e.g., higher screen time, lower physical health activity) (Newlove-Delgado et al., 2021; Viner et al., 2022), reduced student achievement (Hammerstein et al., 2021) and increased risk of child maltreatment (Marmor et al., 2021). Children from lower socio-economic backgrounds suffered disproportionately, and the need to reduce such inequalities is widely recognized (Marmot, 2010, 2014).

Child poverty is associated with poorer physical and mental health, negative educational outcomes and adverse long-term social and psychological consequences (Wickham et al., 2016; Lai et al., 2019). This limits children’s potential development, leading to poor health and life chances in adulthood (Raphael, 2011; Yoshikawa et al., 2012). The relationship is causal: poorer children have worse outcomes in part because they live in low-income households (Cooper & Stewart, 2021). There is also a social gradient, meaning that economic conditions exert influence across the social spectrum (Marmot, 2010). Moreover, longer periods of child poverty have more severe effects, for instance on violent criminality and self-harm in young adulthood (Mok et al., 2018). This all impacts on service demand and expenditure. In England, for example, rising child poverty rates from 2015 to 2020 contributed to over 10,000 additional children entering the care system during that period at an estimated cost of £1.4 billion (Bennett et al., 2022).

Poverty affects child outcomes through different mechanisms. It increases parental stress, which adversely affects inter-parental relationships (e.g., increased conflict, possibly violence) and, in turn, how they parent their children (Family Stress Model; FSM) (Conger et al., 2010). Insufficient income also adversely affects parents’ ability to afford good nutrition, housing, childcare and educational resources, all of which promote child development (Investment Model; IM) (Duncan et al., 2014). These are the two best-known mechanisms but others exist too. For example, there are biological pathways through which co-occurring risks related to poverty (e.g., food insecurity, infectious disease, psychological stress) interact to shape children’s neurocognitive development (Jensen et al., 2017).

Until now, however, prevention and early intervention services have tended to focus their efforts to improve child outcomes on enhancing inter-parental relationships and parenting skills (e.g., via relationship skills education, home visiting, parenting programs, family therapy) or child language, social-emotional and life skills (e.g., early childhood education, school-based programs, youth mentoring). Family interventions often target low-income neighborhoods or low-income families but rarely address poverty *directly*. Instead, they aim to ameliorate its impact *indirectly* by targeting mediating factors (e.g., family conflict, child maltreatment, home learning environment) (Donkin et al., 2014).

By way of illustration, neither the Blueprints (US) nor the Early Intervention Foundation Guidebook (UK) registries of evidence-based programs (EBPs) lists financial outcomes, and very few included programs are categorized as explicitly targeting income or related factors (e.g., financial stress).^1^ Similarly, despite evidence that family conflict is more prevalent in families who experience poverty, programs to address inter-parental conflict focus much more on relationship education and conflict resolution than on reducing economic pressure (e.g., Acquah et al., 2017).

A similar situation pertains in social work, one setting for early intervention. Here, the focus is often on managing individual risk detached from socio-economic conditions (Morris et al., 2018), with practitioners unconsciously investing in an underclass discourse or fearing that it is stigmatizing to discuss a link between poverty and child maltreatment (ibid.). Poverty is invariably the context for interventions but never their target – the “wallpaper of practice: too big to tackle and too familiar to notice” (ibid., p.370). In our experience, there is some economic support activity in other prevention and early intervention services (e.g., via children’s centers in the UK), but it is limited and ad hoc.

## The Case for Refocusing

There is much to be said for interventions that address issues such as early learning, parent mental health, parent substance use, family conflict and parenting. Reviews have identified substantial evidence for the effectiveness of several such home visiting, early years education, parenting support and family therapy programs in improving child social, emotional, cognitive and behavioral outcomes (e.g., Asmussen et al., 2016, 2022). It is likely that some of these psychosocial interventions also improve families’ economic circumstances *indirectly*, although this is rarely measured. The best EBPs are proven in varied contexts with different populations and have extensive materials, training packages and technical assistance, aiding implementation.

However, the limitations of such interventions are also well-known. One is that too many produce null or equivocal results when tested in experimental studies (Axford et al., 2022). Even when they produce positive outcomes, effect sizes are often small, short-term and difficult to replicate (Gillies et al., 2017). There may be good reasons for this, such as the improved design, conduct and reporting of trials, and services as usual—the default control—getting better over time (Axford et al., 2022). Moreover, small effects generated by universal interventions can have more population relevance than larger effects from targeted interventions (Greenberg & Abenavoli, 2017; Tanner-Smith et al., 2018). Even so, we should not be complacent. One avenue to enhance the effectiveness of prevention and early intervention efforts is to focus more on how psychosocial and educational interventions can improve families’ economic circumstances. This might be through adding economic components to them or collaborating more with existing financial and material support services. There are at least three arguments for this refocusing.

First, it is short-sighted and arguably unethical to focus on individual risk without acknowledging or seeking to address (where relevant) the environment in which families live or the social, political and economic contexts that create economic inequalities (Gillies et al., 2017; Featherstone et al., 2018; Morris et al., 2018). For example, services to prevent or intervene early with child abuse and neglect often target parenting behavior only and ignore poverty (Haworth et al., 2022). This is despite good evidence that low income contributes significantly to child abuse and neglect, whether through increased family conflict, domestic violence, parent substance misuse and mental health problems or because parents lack money to adequately feed, clothe, house and generally look after their children (Bywaters et al., 2022). By not viewing poverty as core business, therefore, services can appear to “translate ‘public issues’ into private troubles” (Featherstone et al., 2018: 12–13), implying that parents in those circumstances choose to engage in risky or troublesome behavior and neglect their children.

Second, low income makes it harder for families to engage with other forms of psychosocial support. For example, a meta-analysis of trials of the Incredible Years parenting program found that low socio-economic status (SES) reduced attendance by 8–19% depending on the SES marker (Berry et al., 2022).^2^ This matters because limited intervention effectiveness is sometimes attributed to low engagement in interventions, especially by so-called ‘hard-to-reach’ families (Pote et al., 2019). Recommended strategies to improve service accessibility include providing free childcare, meals and transport—goods in kind—or financial incentives (e.g., Finan et al., 2018; Gonzalez et al., 2018). But if money is sometimes the barrier to parent engagement, so too are the psychosocial implications of being poor and subject to scrutiny and investigation by children’s services. This can leave parents feeling blamed, stressed, ashamed, stigmatized, isolated and disempowered (Featherstone et al., 2018; Gupta, 2018; Gupta et al., 2018). Strengthening families’ economic security would arguably help parents to engage in interventions that support parenting and family relationships.

Third, there is evidence that increasing household income works. A recent systematic review found that it had a positive causal effect on child outcomes, especially for low-income families (Cooper & Stewart, 2021). For instance, a US$1000 (year 2000) annual change in income produced small but non-negligible effect sizes for cognitive (5–37%), social-behavioral (3–22%) and health (1–24%) outcomes in children.^3^ These are comparable to those calculated for low-income families from early interventions, including early education. Household income increases also improved intermediate outcomes that are important for child development (e.g., maternal mental health, parenting, home environment) (ibid.). Similarly, another systematic review concluded that socio-economic interventions (e.g., income supplements, cash transfers, housing support) targeting low-income households can reduce exposure to adverse childhood experiences (Courtin et al., 2019). Effect sizes were strongest for childhood victimization and substance use, with a moderate to high effect size for exposure to domestic violence, and moderate effect sizes for adverse parenting, household mental illness and child maltreatment and neglect.^4^

## Changing Practice

Strategies to prevent or reduce child poverty and its consequences commonly involve early childhood care and education, income redistribution through the benefit and tax systems, and policies to increase employment and the wages of families in poverty (Wickham et al., 2016). The evidence suggests that national policy-level initiatives like these are likely to be effective (ibid.). There is also a movement to ‘poverty-proof’ policies, processes and practices to avoid inadvertently undermining rather than supporting families (Bywaters et al., 2022). A good example is helping schools to ensure that the costs of uniform or extracurricular activities and the administration of free school meals do not exclude or stigmatize poorer children (Mazzoli Smith & Todd, 2016).

However, it is increasingly recognized that practice-based initiatives have a role to play. With a view to reducing the need for children to enter care, a recent review of services in the UK called for greater investment in income maximization services, devolved budgets to allow social workers to give families direct financial or material support, and linking families to other sources of assistance (e.g., loans, foodbanks) (MacAlister, 2022). The Scottish Government (2022) strategy to tackle child poverty also advocates income maximization alongside housing, social security and employment reforms. And in social work, the “poverty-aware” practice movement urges practitioners to better understand the implications of poverty and work with service users to protect their rights, address micro aggressions (everyday experiences of shame and humiliation) and engage in advocacy (e.g., to reduce debts) (Krumer-Nevo, 2016).

There are challenges to scaling up practice-based economic support initiatives and reasons to be cautious; knowledge about their implementation and effectiveness is relatively thin. For example, there is evidence that co-located welfare rights advice in healthcare can improve recipients’ financial circumstances (often substantially) through improved and more stable household income (e.g., backdated payments from unclaimed benefits, successful applications for eligible benefits) and increased confidence in managing finances (Reece et al., 2022). Improvements in clients' physical and mental health and well-being are also reported (ibid.; McGrath et al., 2021; Young & Bates, 2022), although mechanisms driving impact are unclear. Associated gains are reported in nutrition, housing conditions, stress levels and close relationships, and there is some evidence of reduced workload for primary and secondary care services, resulting in cost savings (Reece et al., 2022). The evidence of impact on health is somewhat inconsistent and of limited quality, however, with a need for more experimental research involving larger samples and greater exploration of factors conducive to optimal service delivery. Moreover, there is comparatively little rigorous research on whether and how such services affect (i) parent-child interactions and parenting, and (ii) child physical and psychosocial outcomes. For instance, a systematic review of the impacts of income maximization models in healthcare on families with children aged 0–5 years in high-income countries concluded that there was insufficient evidence to evaluate service effectiveness (Burley et al., 2022).

Meanwhile, qualitative evidence from interviews with social workers and clients indicates the potential and challenges of devolved budgets (Saar-Heiman & Krumer-Nevo, 2021; Westlake et al., 2022). The money is typically used to meet financial and practical needs (e.g., furniture, food, clothing, rent) and help parents develop skills (e.g., driving lessons, tutoring) or obtain therapeutic support (e.g., counselling). Perceived positive impacts include resolving crises, preventing child entry to care and strengthening the family-worker relationship. The latter derives in part from families feeling listened to and understood. That said, tensions can arise regarding who owns the money and how it is spent—such support is relational not a technocratic fix— and, again, more rigorous research on effectiveness is needed.

## Next Steps

**Fig. 1:**
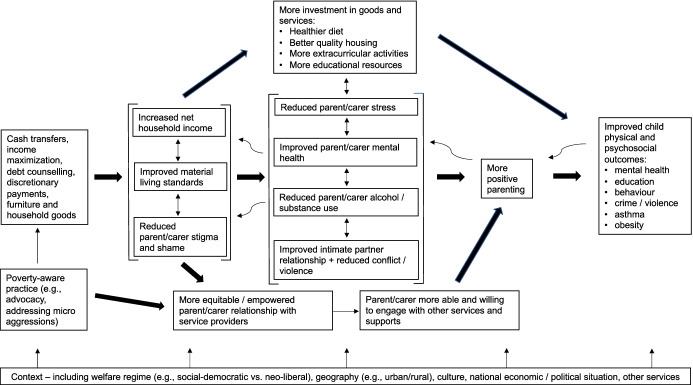
Theorized contribution of financial/material support to family functioning and child outcomes

Prevention and early intervention practice needs to attend more to families’ economic circumstances. This requires several parallel streams of work. First, we need to develop theory. As a start, and drawing on the FSM and IM models, we outline a simple consolidated model showing how financial and material support—delivered programmatically as part of broader service system transformation (e.g., Weiner et al., 2021)—could help to improve family functioning and children’s physical and psychosocial outcomes (Fig. 1).^5^ This needs refining through deeper exploration of the literature, stakeholder and public consultation and effectiveness studies. Second, researchers, providers and practitioners need to co-produce or adapt economic support interventions (or components) targeting low-income families. These might include money advice and management, debt counselling or providing cash or goods. They could be integrated into home visiting, early education, parenting and family therapy programs, or exist as standalone services with signposting or referral by such programs. Third, we must test the implementation, reach and effectiveness of such interventions. Studies should measure participants’ economic circumstances at baseline, post-test and longer-term follow-up, test for moderating effects of the depth and duration of low-income episodes (e.g., acute vs. chronic), explore mechanisms of impact, consider how the effectiveness of family economic support varies between welfare regimes (e.g., social-democratic vs. neo-liberal) and examine which factors best contribute to successful delivery and acceptability to users. The results will have important implications for intervention design.

Addressing economic hardship is no silver bullet; even after controlling for low income, other factors contribute to poor outcomes such as violent offending and substance misuse (e.g., Sariaslan et al., 2014), indicating the need for interventions to target a wider range of familial risk besides merely parental income. Without relevant action, however, services will arguably face an uphill struggle to alleviate the negative impact of inadequate family resources (Cooper & Stewart, 2017). Improving child outcomes requires a joined-up approach – both improving parental capacity *and* reducing the pressure on families caused by poverty (Eisenstadt & Oppenheim, 2019). Investigating how this is done, and potential synergies between the two, is a pressing task.

## Footnotes

1 In Blueprints, 11 (of 105) programs target low socio-economic status (‘family’ category of risk factors), 1 targets parental unemployment (‘family’) and 2 target extreme economic disadvantage (‘community/neighbourhood’). Three programs target > 1 of these risk factors, so in total 12 programs target ≥ 1 of them (11.4%). Risk and protective factors are not listed in the Guidebook.

2 A 16% reduction using the ‘low income’ marker.

3 Standard deviation change based on the RCTs and quasi-experimental studies (n = 19).

4 Other effect sizes were also reported.

5 Delivered independent from, alongside (with signposting and referral) or integrated (as components) into family-orientated psychosocial interventions. It is reasonable to hypothesize that the effects of both types of intervention will be greater when both are active (i.e., they are mutually reinforcing).
